# Tinkering with Tuberculosis

**Published:** 1923-05

**Authors:** 


### TINKERING WITH TUBERCULOSIS.

THE public expenditure in this country on the various special forms of treatment of tuberculosis amounts at the present time to some 2¾ millions annually, out of the rates and the public exchequer. Considered as expenditure on curative treatment, it is largely ineffective because, for one thing, it is hopelessly inadequate, and yet it has come to a halt, so far as State-aided expenditure is concerned, and there is little likelihood of Councils increasing their provision in the absence of further Exchequer assistance. Considered as expenditure on preventive treatment, it is sufficient to say that it is to a large extent expenditure in the wrong direction.

### The Folly of Extravagant Promises.

When the National Insurance Bill was before Parliament and the country in 1911, it was unfortunately carried along to no small extent by the most extravagant promises in regard to the treatment of the Great White Scourge—promises which a simple sum in arithmetic ought to have shown to be utterly incapable of fulfilment. The sum of 1s. 3d. per insured person per annum, made available for the treatment of tuberculosis, was earmarked as to 6d., before the Act came into operation, to provide part of the remuneration of the panel doctor, who had the responsibility for the home treatment of tuberculosis, as for other diseases amongst the insured. The remaining capitation sum of 9d. produced an amount so small that to deal with the applications for institutional treatment, in the light of the expectations created in the minds of the insured persons and their friends, led to a tinkering with cases all along the line.

### The Pound for Pound Basis.

It is true that concurrently with the coming into operation of the Insurance measure County and County Borough Councils were stimulated to make provision, and to embark upon what were optimistically called comprehensive schemes of treatment for the whole population of their area, being encouraged to this end with grants from the Exchequer in aid of approved capital expenditure, and grants in aid of yearly expenditure out of revenue, on the basis of one pound from the Exchequer for every pound spent out of the rates The National Health Insurance Committees, not being in a position to provide treatment—whether by the erection or leasing of institutions or otherwise—but merely to arrange for treatment by contracting with other parties, were recommended to enter into agreements with the Councils, and to hand over the available insurance funds in return for a certain reservation of beds for insured persons. The pound for pound basis then applied to the Council's expenditure over and above the sum so contributed from Insurance funds.

### Responsibility of the Councils.

The limitation of the Insurance Committees' powers to which we have referred gave, from the outset, an air of unreality to their functioning, so far as the treatment of tuberculosis was concerned, and was a source of embarrassment to them, because of the great expectations which had been aroused. In so far as the promises of 1911 were based on the provision actually secured for the insured by the funds earmarked under the Act, they were, as we have said, incapable of fulfilment. In so far as they were based on the probable efforts which the County and County Borough Councils were likely to make, they should never have been made. The Councils persisted for quite a long time in regarding themselves as freed from any responsibility for the treatment of the insured, and in the result the institutional treatment provided in most areas for the insured, even by the most progressively-minded of the Councils, was altogether inadequate, and for the uninsured too often negligible. And the much-heralded provision of treatment of tuberculosis among the insured was dropped, as a National Health Insurance provision, in 1920, with scarcely a protest and with a lack of public interest in the question which was deplorable. The County and County Borough Councils now have the sole responsibility in this matter, assisted, it is true, with special exchequer grants based on past expenditure on the treatment of the insured, but they remain under no illusions as to the existence of other bodies on to whose shoulders they can shift their responsibility, and with their enthusiasm for developments damped by the Minister of Health aided and abetted by Sir Eric Geddes.

### A Royal Commission Needed.

What do the Councils propose to do about it all? Is it not time that the whole question of the public provision of treatment of tuberculosis were fully and frankly faced by the appointment of a Royal Commission with a very wide reference? Have the various forms of treatment justified themselves by results? Have the sanatoria effected cures of patients or even restoration of working capacity to a sufficient degree to permit of their continuance as a form of public curative treatment? Or is the contention fairly made in some quarters that the mortality among sanatorium patients does not show any improvement on that of cases treated in pre-sanatorium days? How far is their cost justified from the point of view of the educational value to the patient, and can this not be equally well effected at much less cost? Is the object of the isolation of advanced cases—admittedly a public health measure—defeated by the too late admission of patients or their refusal to stay in an institution, or both? Is the main function of the dispensary—that of a centre for advice and consultation—being departed from so that general practitioners are relieved of responsibility for the treatment of their patients? Are the experiments of village settlements, or training centres, proving their value, or is it a fact that tuberculous patients are loth to separate themselves from their fellows and settle in colonies, however much it may be demonstrated that that way health and life lie ? Is it not possible to give men suitable sheltered employment at centres while living under hygienic conditions in their own homes ?

### The Question of Employment.

This last question is one to which we believe far too little consideration has been given, and yet it is of first importance. Training in suitable employment while under medical supervision in a sanatorium is an aspect of institutional treatment which was too long almost entirely ignored, but has in recent years received some attention at the hands of the authorities. But what of the patient—for such he must still remain—after he leaves the institution ? The offer of residence in a Tuberculosis Colony, admirably conceived as the idea is, arouses little or no enthusiasm, and will in general be rejected. Is it not possible to provide tuberculous men with sheltered employment while they continue to reside in their own homes, of course under hygienic conditions, and subject to continuous medical supervision ? Will the Trade Unions be prepared frankly to recognise that these men are not economic units, and to co-operate whole-heartedly in schemes for their employment ?

### Prevention the First Object.

We do not pretend that any of these questions would be answered in the same way by competent observers, still less by enthusiasts ; nor do wes profess to answer them ourselves. We should regret any suggestion that we were unfairly ignoring the difficulties with which the whole question is surrounded, or that we failed to recognise that the Nation's conscience has been aroused in recent years in regard to this problem. The National Health Insurance Act undoubtedly focussed public attention on this grave question. The Councils were undoubtedly stimulated to move in the matter. Also, the whole of the last twelve years (including the years of war, when people merely " carried on " in these and other matters) can fairly be regarded as years of experiment. But unquestionably the time has now come to examine the results, to see that, on the best advice obtainable, expenditure shall proceed not only according to plan, but the best plan ; to ask ourselves fairly—Are cases of tuberculosis being manufactured more rapidly than they are being cured, even using that expression with a very restricted meaning ? Expenditure on the public health must not be confused with expenditure on the relief of the individual sufferer, whatever well-meaning enthusiasts may say or think, and expenditure of the latter kind cannot be justified while neglect of insanitary areas and of other sources of manufacture of tuberculosis is so obvious and universal. There must be a close limitation and definition of public expenditure on the treatment of tuberculosis, and the utmost possible expenditure on the attack on the disease everywhere at its source. But first there must be inquiry, and to be effective it must be a patient and prolonged inquiry by a Commission with a very full and wide reference.

